# Aliskiren in Patients Failing to Achieve Blood Pressure Targets With Angiotensin Converting Enzyme Inhibitors or Angiotensin Receptor Blockers

**DOI:** 10.4021/cr201w

**Published:** 2012-07-20

**Authors:** Elizabeth B. Hawkins, Hua Ling, Tammy L. Burns, Aryan N. Mooss, Daniel E. Hilleman

**Affiliations:** a Department of Pharmacy Services, Creighton University Medical Center, Omaha, NE, USA; b Creighton University Cardiac Center, Creighton University School of Medicine, Omaha, NE, USA; c Department of Pharmacy Practice, Creighton University School of Pharmacy and Health Professions, Omaha, NE, USA

**Keywords:** Aliskiren, Angiotensin converting enzyme inhibitors, Angiotensin receptor blockers, Hypertension

## Abstract

**Background:**

To assess the efficacy of aliskiren in patients failing to reach blood pressure (BP) goals with angiotensin converting enzyme inhibitor (ACEI) or angiotensin receptor blocker (ARB).

**Methods:**

A total of 107 patients who failed to reach BP goals on ACEI or ARB were switched to aliskiren. Changes in BP were determined during maximal ACEI, ARB, or aliskiren therapy.

**Results:**

Mean reduction in sBP and dBP with ACEI was 8.5 ± 6.3 mmHg and 6.0 ± 4.7 mmHg, respectively. Mean reduction in sBP and dBP with ARB was 8.3 ± 6.7 mmHg and 5.0 ± 5.2 mmHg, respectively. Mean reduction in sBP and dBP with aliskiren 150 mg/d was 6.7 ± 5.4 mmHg and 5.4 ± 4.8 mmHg, respectively. Mean reduction in sBP and dBP with aliskiren 300 mg/d was 8.6 ± 6.3 mmHg and 6.0 ± 4.9 mmHg, respectively. BP reductions between ACEI, ARB, and aliskiren were not significantly different.

**Conclusions:**

Aliskiren is ineffective in patients failing ACEI or ARB therapy. Given the label changes restricting the use of aliskiren in combination with ACEI and ARB, excess cost compared to ACEI and ARB, and a paucity of outcome data, there is a limited role for aliskiren in practice.

## Introduction

Activation of the renin-angiotensin-aldosterone system (RAAS) plays an important role in the development of end-organ damage in a variety of cardiovascular diseases such as hypertension, heart failure, myocardial infarction, diabetes, and renal disease [1, 2]. Angiotensin converting enzyme inhibitors (ACEI) and angiotensin receptor blockers (ARB) modulate RAAS activity which lowers blood pressure (BP) and have been shown to have a favorable effect on target organ damage [3, 4].

Aliskiren is the only direct renin inhibitor to be approved for use in the United States [5]. It is only indicated for the management of hypertension [6]. The recommended starting dose of aliskiren is 150 mg once daily. In patients whose BP is not adequately controlled at that dose, the daily dose may be increased to 300 mg. Doses above 300 mg/d do not produce further BP reductions, but results in an increased frequency of side effects [5, 6].

A recent Cochrane database review found that aliskiren has a dose-related effect on BP greater than placebo with a BP effect similar to ACEI and ARB [7]. Aliskiren has not been shown to reduce target organ damage alone or in combination with other therapy. Based on a recent FDA warning and contraindication, aliskiren should not used in combination with ACEI or ARB in patients with diabetes or renal dysfunction (creatinine clearance < 60 mL/min) [8]. The fixed-dose combination of aliskiren and valsartan was removed from the United States market.

As a result, aliskiren is generally recommended only for patients that fail to respond to or cannot tolerate an ACEI and/or an ARB. This largely results from the higher cost of aliskiren compared to these agents and due to the lack of outcome data with aliskiren. There is, however, no published data demonstrating the effectiveness of aliskiren in patients failing to achieve BP goals with an ACEI or ARB. The purpose of this study was to evaluate the efficacy of aliskiren in patients failing to reach BP goals with either ACEI or ARB.

## Methods

This study was a retrospective medical record review. It was approved by the Creighton University Institutional Review Board.

### Patients

Patients with hypertension who failed to reach their BP goals despite treatment with adequate doses of ACEI or ARB after a minimum of 8 weeks of therapy were screened for inclusion in the analysis. Patients failing ACEI or ARB therapy secondary to adverse effects were excluded. Adequate doses of ACEI or ARB used in this study are listed in Table 1. Patients failing to achieve their BP goal on ACEI or ARB therapy had that therapy discontinued and were switched to aliskiren 150 mg/d for a minimum of 4 weeks. Patients failing to achieve their BP goal on aliskiren 150 mg/d received 300 mg/d for a minimum of 4 weeks. Patients were allowed to receive other antihypertensive therapy, other than ACEI or ARB, as long as that therapy was not altered at anytime during the study timeframe. Patients were excluded if any of the following conditions were present: renal dysfunction (serum creatinine > 3.0 mg/dL), serum transaminase (ALT or AST) levels > 2 times the upper limit of normal, a known secondary cause of hypertension, or a history of myocardial infarction (MI), acute coronary syndrome, coronary revascularization or stroke in the 6 months preceding the use of ACEI or ARB.

**Table 1:** Minimum Doses of ACEI or ARB Required for Study Inclusion

ACEI	Adequate Daily Dose (mg)	ARBs	Adequate Daily Dose (mg)
Enalapril	20	Candesartan	16
Fosinopril	20	Irbesartan	300
Lisinopril	20	Losartan	100
Ramipril	10	Olmesartan	20
Trandolapril	4	Telmisartan	40
		Valsartan	160

### BP goals

BP goals for patients with uncomplicated hypertension were a systolic BP (sBP) < 140 mmHg and/or a diastolic BP (dBP) < 90 mmHg. BP goals for patients at high risk of coronary artery disease (diabetes mellitus, chronic kidney disease, carotid artery disease, peripheral arterial disease, abdominal aortic aneurysm, or a 10-year Framingham risk score > 10%), stable angina, unstable angina/non-ST elevation MI, or ST segment elevation MI were a sBP < 130 mmHg and/or a dBP < 80 mmHg. BP goals in patients with left ventricular dysfunction or symptomatic heart failure were a sBP < 120 mmHg and/or a dBP < 80 mmHg.

### Data analysis

The absolute change in sBP and dBP was calculated with initial ACE and ARB therapy and compared to that achieved with aliskiren therapy. The proportion of patients who successfully achieved BP goals with aliskiren therapy was also determined. The type and frequency of adverse events documented in the medical record with aliskiren therapy were identified. A paired student t-test was used to assess whether the mean change in sBP and dBP was significantly different between ACEI or ARB therapy and aliskiren therapy. A P-value of < 0.05 was considered statistically significant.

## Results

One hundred and seven patients were included in this study. The average age of patients was 69 ±11 years with the majority being white (84%) and male (54%) with an average serum creatinine of 1.3 ± 0.8 (Table 2). However, only 6% of patients had a serum creatinine > 2.0 mg/dL at baseline. In addition to hypertension, a substantial number of patients had a variety of cardiac and non-cardiac comorbidities.

**Table 2:** Comorbidities in the 107 Study Patients

Cardiac History	
HTN	107 (100%)
MI	34 (32%)
Angina	21 (20%)
PCI/CABG	17 (16%)
HF	11(10%)
PAD	4 (4%)
VHD	3 (3%)
Stroke	3 (3%)
Hyperlipidemia	67 (63%)
Arrhythmias	14 (13%)

Non-Cardiac History	
Diabetes mellitus	38 (36%)
Thyroid	32 (30%)
Psychiatric	17 (16%)
GI Disease	10 (9%)
Arthritis	11(10%)
COPD/Asthma	11(10%)
Renal disease	6 (6%)

Of the 107 patients included in this study, 79 patients failed to reach BP goals during treatment with ACEI and 28 patients failed to reach BP goals during treatment with ARB. The average doses and duration of ACEI or ARB therapy are summarized in Table 3. All but 6 of the 107 patients received additional antihypertensive therapy in combination with ACEI or ARB therapy that was continued during aliskiren therapy (Table 4). All 6 patients that received monotherapy had failed ACEI therapy. All patients were switched to aliskiren within 7 days of stopping ACEI or ARB therapy (Table 5). The average duration between discontinuation of ACEI or ARB therapy and initiation of aliskiren therapy was 4.2 ± 1.7 days.

**Table 3:** Treatment Failure With ACEI or ARB Prior to Aliskiren Therapy

ACEI				ARB			
Drug	No of pts	Average Dose (mg)	Average Duration of Therapy (weeks)	Drug	No of pts	Average Dose (mg)	Average Duration of Therapy (weeks)
Lisinopril	28	22.5	14.1	Candesartan	6	21.3	13.0
Enalapril	8	20.0	15.5	Olmesartan	10	30.0	12.1
Fosinopril	16	28.8	12.5	Telmisartan	3	66.7	12.7
Ramipril	15	11.3	14.4	Valsartan	2	240.0	12.0
Trandolapril	12	8.0	13.3	Irbesartan	5	100.0	11.0
				Losartan	2	300.0	12.6
Total	79				28		

**Table 4:** Additional Therapies Used in Combination With an ACEI or ARB and Aliskiren

Additional antihypertensive therapy	ACEI (n = 73)	ARB (n = 28)
Hydrochlorothiazide (HCTZ)	27	12
Beta-blocker (BB)	20	5
Calcium channel blocker (CCB)	18	6
HCTZ plus BB	4	2
BB plus CCB	3	2
BB plus CCB plus HCTZ	1	1

**Table 5:** Time Between Discontinuation of ACEI or ARB Therapy and Initiation of Aliskiren Therapy

Days between therapy	1	2	3	4	5	6	7	8	9
Number of patients	2	12	23	43	6	6	10	3	2

The mean reduction in sBP and dBP with ACEI was 8.5 ± 6.3 mmHg and 6.0 ± 4.7 mmHg, respectively (Table 6). The mean reduction in sBP and dBP with an ARB was 8.3 ± 6.7 mmHg and 5.0 ± 5.2 mmHg, respectively. Three of the 79 patients receiving ACEI therapy reached a BP < 140/90 mmHg, but all three of these patients had BP targets < 130/80 mmHg. One out of the 27 patients receiving ARB therapy reached BP < 140/90 mmHg, but this patient also had a BP target < 130/80 mmHg.

**Table 6:** BP Response to ACEI or ARB Therapy

	ACE (n = 79)	ARB (n = 28)	Total (n = 107)
Baseline BP (mmHg)	165.4 ± 10.7 103.3 ± 5.2	166.4 ± 10.3 102.7 ± 4.8	165.7 ± 10.6 103.1 ± 5.1
Change in BP (mmHg)	↓8.5 ± 6.3 ↓6.0 ± 4.7	↓8.3 ± 6.7 ↓5.0 ± 5.2	↓8.4 ± 6.4 ↓5.8 ± 4.9
Final BP (mmHg)	156.9 ± 10.3 97.3 ± 4.9	158.1 ± 9.9 97.7 ± 5.1	157.3 ± 10.2 97.1 ± 5.0

The mean reduction in blood pressure with aliskiren 150 mg per day after an average of treatment duration of 8.4 weeks was 6.7 ± -5.4 mmHg sBP 5.4 ± 4.8 mmHg dBP versus 300 mg per day for an average of 8.8 weeks was 8.6 ± 6.3 mmHg SBP 6.0 ± 4.9 mmHg dBP (Table 7). The differences in the magnitude of BP reduction between ACEI/ARB and aliskiren were not significantly different. Of the 107 patients, only 1 (< 1%) patient achieved their BP goal during aliskiren therapy (< 130/80 mmHg).

**Table 7:** BP Response to Aliskiren Therapy

BP Response to Aliskiren (n = 107)		
	Mean BP (mmHg)	Mean Change in BP
Baseline BP (mmHg) (includes background therapy)	161.8 ± 10.8 101.7 ± 6.3	
150 mg per day/8.4 wks	155.1 ± 11.3 96.3 ± 6.8	↓6.7 ± 5.4 ↓5.4 ± 4.8
300 mg per day/8.8 wks	153.2 ± 10.9 95.7 ± 6.9	↓8.6 ± 6.3 ↓6.0 ± 4.9

No dose limiting side effects were documented during aliskiren therapy. No patient experienced hyperkalemia, a substantial increase in serum creatinine (> 0.05 mg/dL), rash, or gastrointestinal disturbances. Four patients (4%) did experience adverse effects with aliskiren which included headache, dizziness, nasal congestion, and paresthesias.

## Discussion

As a direct renin inhibitor, aliskiren represents a novel addition to current antihypertensive therapy and an alternative way of impacting the RAAS. A theoretical advantage of blocking the RAAS early in the pathway included inhibition of reflex renin activity induced by downstream RAAS blockers [9]. It has also been theorized that aliskiren would be less likely to produce increases in angiotensin II levels and to stimulate aldosterone escape [10].

Aliskiren was approved by the FDA for use in the United States in 2007 [5]. Aliskiren monotherapy has been shown to be superior to placebo in a number of short-term clinical trials in patients with uncomplicated hypertension [11]. Aliskiren monotherapy has also been shown to be as effective in lowering BP compared to a number of other monotherapy antihypertensive agents including thiazide-like diuretics, ACEI, ARB, and calcium channel blockers [11]. A recent Cochrane database review found that aliskiren has a dose-related BP lowering effect greater than placebo with a magnitude of BP lowering similar to ACEI and ARB [7].

A comprehensive research program was implemented to identify the potential of aliskiren to reduce target organ damage for indications similar to ACEI and ARB. One important limitation that impacted the assessment of aliskiren was that ACEI or ARB therapy reduces target organ damage and/or mortality in patients with MI, heart failure, and nephropathy [12]. Their use in these disease states is considered a class I indication [12]. As a result, aliskiren had to be tested against background therapy including either ACEI or ARB therapy. Outcome studies with aliskiren compared it against placebo in patients already receiving ACEI or ARB therapy. The net result was that the aliskiren outcome studies were testing combinations of RAAS modulators compared to a single RAAS modulator.

The Aliskiren in Left Ventricular Hypertrophy (ALLAY) study compared 9 months of treatment with aliskiren 300 mg/d, losartan 100 mg/d, and a combination of the two drugs in 465 patients with hypertension, increased ventricular wall thickness, and a body mass index > 25 kg/m^2^ [13]. Other antihypertensive therapy could be added to achieve BP control. All three treatment groups experienced similar reductions in BP. Cardiac magnetic resonance imaging was performed at baseline and at the end of study to assess left ventricular mass index. Aliskiren was found to be as effective as losartan in reducing left ventricular mass index (P < 0.001 for non-inferiority). However, the combination of aliskiren and losartan was no more effective in reducing left ventricular mass index than either monotherapy (P = 0.52).

In the Aliskiren in the Evaluation of Proteinuria in Diabetes (AVOID) study, 599 patients with hypertension, type 2 diabetes, and nephropathy received open-label losartan 100 mg/d for 3 months [14]. Patients were then randomized in double-blind fashion to receive aliskiren 150 mg/d for 3 months followed by aliskiren 300 mg/d for an additional 3 months or to placebo for 6 months. All patients also received losartan 100 mg/d during the 6 month double-blind phase of the trial. The primary study outcome was the reduction in the ratio of albumin to creatinine measured in an early morning urine sample. Compared to placebo, aliskiren reduced the mean urinary albumin-to-creatinine ratio by 20% (P < 0.001). In addition, patients achieving a reduction of 50% or more in the urinary albumin-to-creatinine ratio were observed in 25% of aliskiren patients compared to 12.5% of placebo patients (P < 0.001). Aliskiren therapy did not significantly reduce sBP and dBP compared to placebo (-2 mmHg/-1 mmHg). In this short term trial, there was no increase in serious adverse events including hyperkalemia or acute kidney injury with combination therapy compared to losartan alone. The investigators concluded that aliskiren may have renoprotective effects independent of BP lowering.

In the Aliskiren Observation of Heart Failure Treatment (ALOFT) study, patients with New York Heart Association class II-IV heart failure, a current or past history of hypertension, and plasma brain naturetic peptide (BNP) > 100 pg/mL were randomized to 3 months of treatment with aliskiren 150 mg/d (n = 156) or placebo (n = 146) [15]. All patients had to be receiving an ACEI or ARB plus a beta-blocker. The primary efficacy endpoint was the change in N-terminal pro-BNP (NT ProBNP). NT ProBNP fell by 244 ± 2025 pg/mL during aliskiren therapy and increased by 762 ± 6123 pg/mL during placebo therapy (P = 0.01). The frequency of renal dysfunction, symptomatic hypotension, and hyperkalemia were not different between aliskiren and placebo. Despite the favorable neurohumoral effects of aliskiren, the lack of power to detect hard clinical endpoints and a short duration of follow-up are major limitations of this study.

The Aliskiren and Valsartan to Reduce NT ProBNP via Renin-Angiotensin-Aldosterone-System Blockade (AVANT GARDE)-TIMI 43 study enrolled 1,101 patients with elevated NT ProBNP (> 400 pg/mL) 3 - 10 days after an acute coronary syndrome (ACS) event [16]. Patients with evidence of heart failure or an ejection fraction < 40% were excluded. Patients were randomized to aliskiren 300 mg/d, valsartan 320 mg/d, a combination of aliskiren and valsartan, or placebo. In the combination therapy treatment arm, aliskiren 75 mg/d was added to valsartan at week 4 and up-titrated each week over the next 4 weeks to a maximum of 300 mg/d. The total duration of treatment was 8 weeks. The primary study outcome was the relative change in NT ProBNP from baseline to the end of the 8 weeks of treatment. NT ProBNP levels declined significantly in each treatment group but the rate of decline was not significantly different between groups. The average reduction in NT ProBNP in the aliskiren, valsartan, combination, and placebo groups were 44%, 39%, 36%, and 42%, respectively. In addition, active therapy did not impact the occurrence of cardiovascular death, MI, or hospitalization for heart failure. Serious adverse events were more common with active therapy (14.4%) compared to placebo (9.4%), but were significantly greater than placebo only with the combination of aliskiren and valsartan (16.5%; P = 0.012). This study provides further evidence that early intervention with RAAS modulating drugs in patients with ACS who do not have clinical evidence of heart failure or left ventricular function provides little benefit. It also indicates that combining two RAAS modulating drugs is associated with a greater risk of toxicity compared to the use of a single RAAS modulator.

In the Aliskiren Study in Post-MI Patients to Reduce Remodeling (ASPIRE) study, 820 patients were randomized to aliskiren or placebo within 2 to 6 weeks of an acute MI who had an ejection fraction < 45% [17]. Patients had to be receiving antiplatelet therapy, an ACEI or an ARB, a beta-blocker, and a statin for two weeks prior to randomization. Patients were also allowed to be treated with an aldosterone receptor antagonist. The primary study endpoint was the change in left ventricular end-systolic volume from baseline until the end of the treatment period at 36 weeks. There was no significant difference between aliskiren and placebo in left ventricular end-systolic volume, left ventricular diastolic volume, ejection fraction, or infarct size. Aliskiren was associated with a greater frequency of renal dysfunction (2.4% vs 0.9%; P = 0.09), hypotension (8.8% vs 4.5%; P = 0.02), and hyperkalemia (5.2% vs 1.3%; P = 0.001). The investigators concluded that the use of dual RAAS modulators produced no evidence of benefit but a greater frequency of adverse effects.

In the Aliskiren Trial in Type 2 Diabetes Using Cardiovascular and Renal Disease Endpoints (ALTITUDE), 8,606 patients with type 2 diabetes and renal dysfunction (GFR < 60 mL/min) were randomized to aliskiren or placebo [8]. All patients had to be receiving an ACEI or an ARB. The study was terminated prematurely when the data safety and monitoring board identified a significant increase in adverse renal events, nonfatal stroke, hyperkalemia, and hypotension. These results are similar to the findings of the Ongoing Telmisartan Alone and in Combination with Ramipril Global Endpoint Trial (ON-TARGET) which found no improvement in cardiovascular or renal events and higher rate of kidney injury, hyperkalemia, and hypotension with dual RAAS therapy compared to the use of a single RAAS modulating drug [18]. Following the termination of the ALTITUDE trial, the FDA advised that concomitant use of aliskiren with an ACEI or an ARB is contraindicated in patients with diabetes or renal dysfunction (GFR < 60 mL/min). In addition, the fixed-dose combination of aliskiren and valsartan has been removed from the market in the United States.

Aliskiren is not superior to ACEI or ARBs in the management of hypertension and has not been shown to reduce target organ damage in patients who require the use of ACEI or ARB. Aliskiren increases kidney injury, hyperkalemia, and hypotension when combined with another RAAS modulating drug in patients with diabetes or renal dysfunction. Our single center experience suggests that aliskiren is not effective in patients that have failed to reach their BP goals following adequate doses and durations of therapy with ACEI or ARB. These findings should be validated by a larger prospective study before an absolute recommendation can be made about the use of aliskiren in this patient population. Current evidence suggests that the role of aliskiren in the management of hypertension is limited. Aliskiren use should be limited to those patients who are unable to tolerate ACEI or ARB therapy.
